# Etiology of intellectual disability in individuals from special education schools in the south of Brazil

**DOI:** 10.1186/s12887-020-02382-5

**Published:** 2020-11-04

**Authors:** Luan Freitas Oliveira, Tiago Fernando Chaves, Nathacha Baretto, Gisele Rozone de Luca, Ingrid Tremel Barbato, Jorge Humberto Barbato Filho, Maristela Ocampos, Angelica Francesca Maris

**Affiliations:** 1 Departamento de Biologia Celular, Embriologia e Genética, Universidade Federal de Santa Catarina-UFSC, Florianópolis, SC 88040-900 Brazil; 2 Hospital Infantil Joana de Gusmão, Florianópolis, SC 88025-301 Brazil; 3 Laboratório Neurogene, Florianópolis, SC 88015-440 Brazil

**Keywords:** Intellectual disability, Genetics, Epidemiology, Etiology, Family history, Familial intellectual disability

## Abstract

**Background:**

Intellectual Disability (ID) is characterized by significant limitations that affect intellectual functioning, adaptive behavior, and practical skills which directly interfere with interpersonal relationships and the environment. In Western countries, individuals with ID are overrepresented in the health system, often due to associated comorbidities, and its life-time cost places ID as one of the most expensive conditions of all diagnoses in the International Classification of Diseases. Most of the people affected (75%) live in low-income countries, suffer from malnutrition, lack health care, and do not have access to adequate treatment. The aim of this study was to obtain an estimate of the diagnostic status as well as the prevalence of familial ID among individuals with serious (moderate or severe) ID in a region of the State of Santa Catarina, investigating attendees of special education schools of the Florianópolis Macroregion.

**Methods:**

This was a cross-sectional study conducted between August 2011 and August 2014, through a semi-structured screening questionnaire for the collection of relevant developmental, clinical, familial and educational data, applied in an interview to guardians of students of special education schools of the macroregion of Florianópolis.

**Results:**

The participant special schools enrolled close to 1700 students during the study period and the questionnaire was applied to 849 (50.5%). The male to female ratio of the participants was 1.39:1. Clear etiologic explanations were relatively scarce (24%); most diagnoses referring only to the type and the degree of impairment and for the majority (61.4%) the cause was unknown. About half were sporadic cases within their families (considering three generations). For 44.2% at least one other case of an ID-related condition in the extended family was mentioned, with 293 (34.5%) representing potential familial cases.

**Conclusion:**

Here we describe the epidemiological profile, the available diagnostics, etiology, family history and possible parental consanguinity of participants with ID of special education schools in the South of Brazil. The main results show the need for etiological diagnosis and uncover the relevance of potential hereditary cases in a population where consanguineous unions have a relatively low frequency (0,6%) and highlight the need for public health actions.

**Supplementary information:**

**Supplementary information** accompanies this paper at 10.1186/s12887-020-02382-5.

## Background

Intellectual Disability (ID) is characterized by significant limitations that affect intellectual functioning, adaptive behavior, and practical skills, that interfere with interpersonal relationships and the environment, originated before the age of 18 [1–3]. ID is a permanent condition that affects not only the individual but his whole family. Reliable data on ID prevalence have been shown to vary widely from 0.93/100 to 156/1000 depending on the population and the assessment tools used for each study [4, 5].

In Western countries, people with ID represent a significant cost within healthcare services, possibly because of lower access to healthcare and an increased risk of hospitalization, resulting in a lifetime cost of US $ 1–2 million in Europe and the United States [6]; therefore considered the most costly condition of all diagnoses listed of the International Classification of Diseases (ICD-10) [7], overcoming dementia and cancer [8]. Intellectual disability is unevenly distributed between industrialized and developing countries and the majority (75%) of people affected live in low-income countries, suffer from malnutrition, lack health care, and do not have access to adequate treatment [9]. Meta-analysis studies of the prevalence and incidence of intellectual disabilities such as Maulik (2011) and Mackenzie (2016) demonstrate the scarcity of studies that report the incidence of ID as a whole, but show that the global prevalence of ID stabilized around 1% when considering adaptive abilities [4, 10] and it is estimated that about 85% have mild, 10% moderate and 5% have severe and profound ID [11]. Mild ID is credited mainly to social/cultural/economic factors such as the level of maternal education, access to education, opportunity and access to healthcare and adequate nutrition, and therefore varies much among developing and industrialized countries, whereas the prevalence of severe ID is relatively stable [12].

The causes of ID are quite heterogeneous, as they may be due to factors of genetic, environmental or multifactorial origin [13, 14], however 25 to 60% are attributed to genetic causes [15–17]. The evaluation of the ID traditionally is done through psychometric tests of intelligence, known as an Intelligence Quotient (IQ) test, that nowadays include evaluation of adaptive functioning or life skills [18]. However, in several lower-income countries the possibility to have a formal IQ test is restricted to the wealthier population, therefore in Brazil for most of the individuals the diagnosis of ID and the classification of its severity are done by medical doctors that rely on clinical observations complemented by information given by parents and teachers. The DMS-V sediments this more practical approach to the diagnosis of ID, emphasizing the conceptual, social and practical skills to classify the intellectual performance into mild, moderate, severe and profound without the need of a formal IQ test [18] Although the technologies used to diagnose ID have advanced a great deal in recent years, for 30 to 60% the etiology still remains unclear, which is an even greater concern when the case is familial [19]. The overall impact of familial ID depends greatly on cultural differences, not only related to consanguinity but also to the size of the families (number of children of each generation) [20, 21].

The aim of this study was to obtain an estimate of the diagnostic status as well as the prevalence of familial ID among individuals with serious (moderate or severe) ID in the State of Santa Catarina, investigating attendees of special educations of the Florianópolis Macroregion.

## Methods

This study was a cross-sectional study, conducted between August 2011 and August 2014, through a semi-structured screening questionnaire, elaborated to collect information from the pre-and postnatal phases, child development, family data, family history of three generations (when possible), dysmorphological aspects and information about the development of students from the nine schools of special education maintained by the Associação de Pais e Amigos dos Excepcionais - APAE (Association of Parents and Friends of the Exceptional), as listed: APAE Águas Mornas; APAE Angelina; APAE Anitápolis; APAE Biguaçu; APAE Florianópolis; APAE Palhoça; APAE Rancho Queimado; APAE Santo Amaro da Imperatriz; APAE São José and the Fundação Catarinense de Educação Especial – FCEE (Foundation of Special Education of the State of Santa Catarina). In contrast to the school of Florianópolis, which is in the main city, many are situated in the countryside of the region, up to 70 km from the city, in small urban areas. Those comprise the major schools of special education of the now extinct 18 SDR (18th Regional District) of the Florianópolis Macroregion, with its nucleus composed of the conurbation of Florianópolis with neighboring municipalities, home to about 925,576 people with a population density of 158.6 hab/km^2^, at the time of the study, the largest population in the state of Santa Catarina [22]. The participating special education schools had 1681 students enrolled in the year 2014, most with serious ID (moderate to profound), since individuals with mild ID are typically integrated into the regular school system. The APAE Brasil (Associação dos Pais e Amigos dos Excepcionais - *Association of Parents and Friends of Exceptional People*) is a non-profit philanthropic entity, that maintains schools responsible for the care of people, mostly with serious intellectual disabilities, from 0 years to the end of life, and is present in more than 2 thousand municipalities throughout the national territory [23]. In Brazil these legally are schools in the modality of special needs education, and their attendees are commonly referred to as students.

**Elaboration and application of the questionnaire:** The semi-structured questionnaire used was developed by the multidisciplinary team of the present project, including some questions inspired by a questionnaire that the school of the APAE of São José applies to new school admissions. It contains closed, semi-open and open questions and was answered by one of the parents (or legal guardian) of the enrolled student during an interview, after signing the Free and Informed Consent Form. The families were invited to participate through a note sent home with the students, explaining the aims of the study.

The questionnaires were applied in the school premises either by members of the project team (professors and students of the Neurogenetics Laboratory at the Federal University of Santa Catarina - UFSC) or by teachers from the schools, previously trained by the research team. This training took place individually or in group meetings with the schoolteachers. No information was collected from students whose parents/guardians did not agree to participate in the study.

Besides scheduled meetings during school hours, the presence of the parents/guardians in the school for celebrations or regular parent-teacher meetings was also used as an opportunity to recruit parents and to conduct the interviews to answer the questionnaire. A translated version of the questionnaire can be found as Supplementary material. This study was approved by the Ethics Committee in Research with Human Beings of the UFSC (no 1046/11 Cover: 426223).

**Intellectual disability evaluation in the studied population:** to gain access to these schools the attendees need to have a diagnosis of developmental delay (when under 5 years of age) or a diagnosis of moderate, severe or profound ID. Rather than resulting from a formal intelligence testing, the diagnosis commonly was given by medical doctors relying on clinical assessment, similar as described in the DSM-V. The special needs schools perform a multidisciplinary work which include (when possible) nurses, doctors, speech therapists, physiotherapists, psychologists and teachers, and the individual school records are well compiled with their information. These records were used to complement the questionnaires applied to the parents and/or guardians, which often were individuals that themselves had intellectual shortcomings, known to the school staff. Still, the classification into the different levels of ID severity might not be completely accurate, and for some cases the severity of the ID was not specified.

**Assessment of familial intellectual disability:** Considering three generations (or more), the students were classified according to the family history into: A) Sporadic cases (no other known case of ID in the family); B) Potential familial ID (one or more additional cases of ID in the family, where a common etiology could not be excluded); C) Unlinked ID-related cases (common etiology is excluded, e.g. participant with idiopathic congenital ID and other case(s) in the family due to known cause, like trauma, infection, Down syndrome); D) Possibly familial, however not linked to the participant’s ID (e.g. participant with Down syndrome but two or more additional cases with idiopathic ID in the family); E) Family data missing (e.g. adopted participant or the questionnaire was incompletely answered).

## Results

**Fig. 1:**
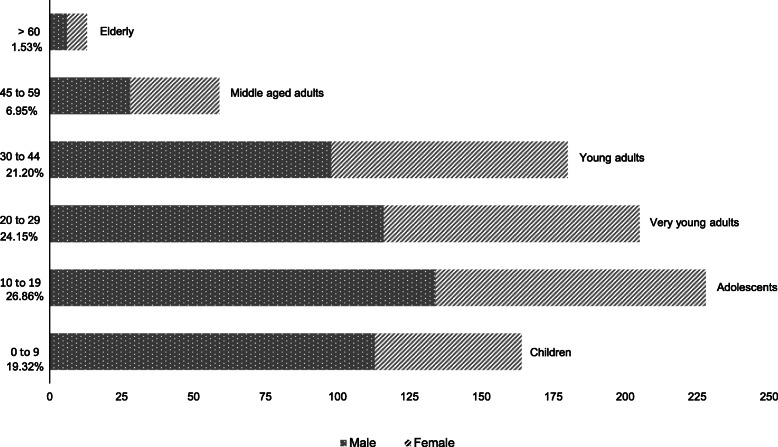
Age group and gender of the participants (*n* = 849)

Of the 1681 students enrolled in the nine special education schools maintained by the APAE plus the FCEE of the 18th SDR, questionnaires were applied to 849 (50.5%). Figure 1 presents the number of participants per age group and gender. The overall male to female ratio was 1.39 M:1F (495 males:354 females) with an age range from 9 months to 66 years, with about 46% from 0 to 19 years of age considered children and adolescents, 52.3% adults and 1.5% elderly, according to the World Health Organization [24].

Table 1 shows the main diagnosis reported for each participant (when the main diagnosis is not ID, the individual usually has comorbid ID, because ID is the reason for acceptance to attend the special schools of this study). The etiologic explanations are relatively scarce, and most diagnoses refer to the degree or main type of impairment of the student and not to the underlying factor or cause, being the majority (61.4%) of unknown primary cause, classified as idiopathic. Among the idiopathic cases, moderate ID was the most prevalent, with almost 1/3 (31%) of all diagnoses, followed by autism (13.5%). Cerebral palsy (11.7%) was excluded from the idiopathic category due to its frequent relation with perinatal hypoxia/anoxia or other trauma/insult, although mostly unknown genetic factors are considered to play a major role [25, 26]. Another difficult to pinpoint category is the one that was named “known conditions of uncertain etiology” (2.6%), because it encompasses known causes of ID, however the etiology of the primary defects (e.g. malformations or seizures, which may result in ID) are largely unknown. Down’s syndrome was the most frequent cause of known genetic etiology, affecting 116 participants, approximately 13.7% of the total. Meningitis was the most common infectious disease-causing ID, cited for 3.7% of the participants, followed by rubella (1.2%) and toxoplasmosis (0.7%).

**Table 1:** Clinical-etiological classification of 849 participants with ID, Florianópolis macroregion, Brazil, 2011–2014

Etiological Diagnosis (%)	Informed Diagnosis	N° of Cases	%	Female	Male
**Idiopathic (61.4%)**	**Idiopathic ID (40.40%)**				
	Unspecific	12	1.4%	6	6
	Mild	24	2.8%	12	12
	Moderate	263	31%	119	144
	Severe	44	5.2%	19	25
	**Autism Spectrum Disorders (14.13%)**				
	Autism	115	13.6%	26	89
	Asperger Syndrome	5	0.6%	1	4
	**Other Brain Disorders (6.83%)**				
	Developmental Delay	21	2.5%	9	12
	Global Developmental Delay	7	0.8%	0	7
	Chronic Encephalopaty	2	0.2%	0	2
	Multiple Deficiency	22	2.6%	12	10
	Epilepsy	6	0.7%	2	4
**Cerebral Palsy (11.8%)**	Cerebral Palsy	100	11.8%	44	56
**Known Conditions of Uncertain Etiology (2.6%)**	Agenesis of the Corpus Callosum	2	0.2%	1	1
	Hydrocephalus	1	0.1%	1	0
	Hypomelanosis of Ito	1	0.1%	1	0
	Microcephaly	4	0.5%	2	2
	Arnold Chiari Syndrome	1	0.1%	1	0
	Dandy Walker Syndrome	1	0.1%	0	1
	Dubowitz Syndrome	1	0.1%	0	1
	Lennox-Gastaut Syndrome	2	0.2%	2	0
	West Syndrome	9	1%	3	6
**Infectious Diseases (6.1%)**	Cytomegalovirus	3	0.4%	2	1
	Meningitis	31	3.7%	16	15
	Rubella (German measles)	10	1.2%	7	3
	Measles	2	0.2%	1	1
	Toxoplasmosis	6	0.7%	4	2
**Trisomy 21 (13.7%)**	Down syndrome	116	13.7%	45	71
**Other Chromosomal Alterations (0.9%)**	Structural or Numerical	8	0.9%	4	4
**Microdeletion and Microduplication Syndromes (0.7%)**	Cri-du-chat Syndrome	1	0.1%	1	0
	Prader-Willi Syndrome	4	0.5%	1	3
	Williams syndrome	1	0.1%	0	1
**Monogenic Causes (2.8%)**	Tuberous Sclerosis	1	0.1%	1	0
	Angelman Syndrome	5	0.6%	3	2
	Allan-Herndon-Dudley Syndrome	1	0.1%	0	1
	Cohen Syndrome	1	0.1%	0	1
	Cornelia de Lange Syndrome	2	0.2%	2	0
	Gorlin-Goltz Syndrome	1	0.1%	0	1
	Kabuki Syndrome	3	0.4%	0	3
	Marinesco-Sjogren Syndrome	2	0.2%	1	1
	Fragile X Syndrome	4	0.5%	1	3
	Rett Syndrome	4	0.5%	4	0
**Total**	–	849	100%	354	495

**Fig. 2:**
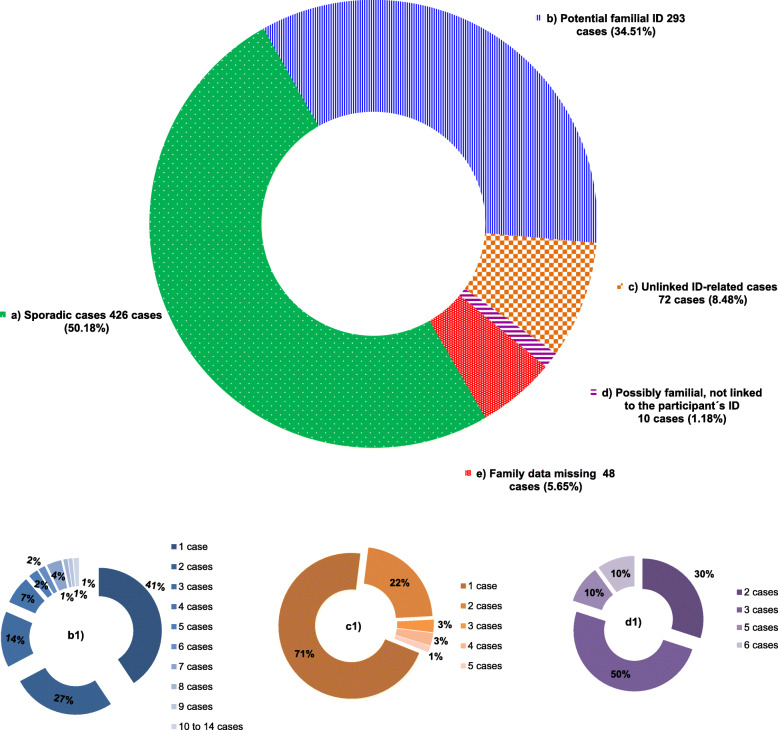
Overview of cases considering the family history of at least three generations. **a** Sporadic cases: participants that, according to available information, have no other ID-related case in the family; **b** Potential familial ID: cases that, according to the questionnaire, could represent hereditary cases; **b1** Percentage of the number of additional possibly related cases in a same family (e.g. 41% of the participants considered a potential familial case reported only one additional case in the extended family); **c** Unlinked ID-related cases: families with probable etiologically unlinked ID-related cases; **c1** Percentage of the number of additional unlinked ID-related cases in each family; **d** Possibly familial, not linked to the participant’s ID: cases of familial ID possibly linked to each other, but not to the cause of the participant’s ID; **d1** Percentage of the number of additional cases in a same family; **e** Family data missing: cases that according to the survey of the questionnaires lacked data to fit into another group (e.g. cases of adoption)

**Fig. 3:**
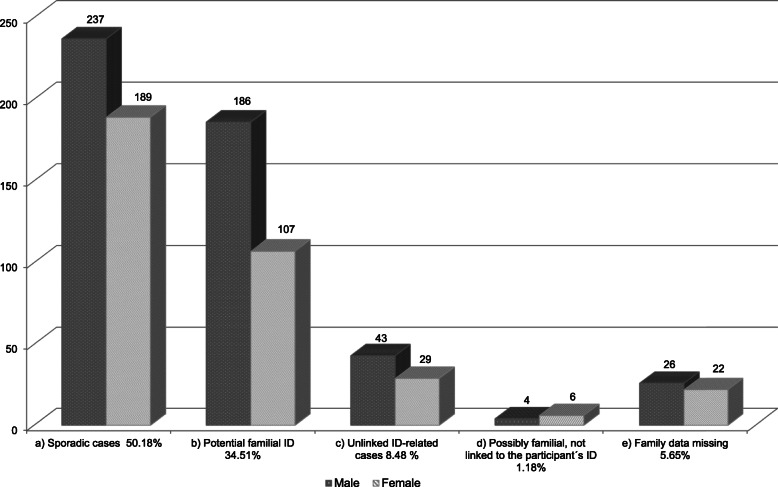
Participants grouped according to family history spanning at least three generations with respect to gender. **a** Sporadic cases: participants that, according to available information, have no other ID-related case in the family; **b** Potential familial ID: participant could represent a familial case; **c** Unlinked ID-related cases: families with etiologically unlinked ID-related cases; **d** Possibly familial, not linked to the participant’s ID: cases of familial DI possibly linked to each other, but not to the cause of the participant’s ID; **e** Family data missing: cases that according to the survey of the questionnaires lacked data to fit into another group (e.g. cases of adoption)

A predominance of males was observed for the autism spectrum disorders (3.4:1), Down syndrome (1.6:1) and idiopathic moderate ID (1.2:1). Regarding familial cases of ID, in general three generations were considered and the participants were classified according to the family history (see methods). Half of the participants 426 (50.2%) reported no other case of ID within their family group, being considered as sporadic cases (Fig. 2a). For 375 (44.2%) at least one other case of an ID-related condition in the family was mentioned (Fig. 2b, c, d). Of these, for 293 participants, which represent 34.5% of all, a common underlying cause could not be ruled out and were, therefore, classified as “Potential familial ID” (Fig. 2b). They were comprised of 186 male participants and 107 female participants (Fig. 3), a male to female ratio of 1.74:1. In these potential familial cases, 41% reported only one additional case in the family, 27% two additional cases and 32% three or more additional cases, the most extreme being a family with 14 additional individuals with ID or ID-linked disabilities (Fig. 2b1). Somewhat unexpected was the considerable number of participants, 72 (8.5% of all) with “Unlinked ID-related cases” (see methods) that reported not only one, but several additional ID cases within the family, where only relying on the questionnaire, a causal relationship among them was ruled out (Fig. 2c). For 10 of participants with two or more additional ID-related cases in the family (1.2% of all), despite them clearly not having the same etiology as the participant’s ID they were possibly familial, linked within each other, e.g. participant with Down syndrome and family members with possible Fragile-X syndrome (Fig. 2d). For 48 (5.7%) participants the family data of the biological parents was unknown (e.g. adopted participant), or the questionnaire was incomplete (Fig. 2e).

One of the questions asked the parents if they were related; 631 participants (74.3%) reported no consanguinity, 34 participants (4%) had related parents and 184 (21.7%) did not know or did not respond (Table 2). Regarding color/ethnic background, most participants were reported as white (80.3%) and most of their parents identified themselves as Brazilians (when the ethnic admixture was high) or descendants of Europeans (Table 2). Parents’ education was also asked for and among the informative answers, it was found that about half of the fathers 47.8% and the mothers 55% did not even have high school education (Table 2).

**Table 2:** Demographic information of research participants (*n* = 849)

Reported consanguinity among the parents of the participants	n		%	
Consanguinity present	34		4	
No Consanguinit	631		74.3	
Unknown	184		21.7	
**Students' skin color statement**^a^				
Yellow	2		0.2	
White	682		80.3	
Black	34		4	
Brown	66		7.8	
Did not answer	65		7.7	
**Reported ethnic background.**	**Paternal**		**Maternal**	
	**n**	**%**	**n**	**%**
European	174	20.5	201	23.7
*German*	*78*	*9.2*	*96*	*11.3*
*Italian*	*58*	*6.8*	*59*	*7*
*Portuguese*	*22*	*2.6*	*32*	*3.8*
*Other European*	*16*	*1.9*	*14*	*1.7*
Asians	3	0.4	3	0.4
Africans	20	2.4	21	2.5
Amerindians	14	1.7	19	2.2
Other Americans	7	0.8	3	0.4
Brazilians^a^	412	48.5	408	49
Did not answer	219	25.8	194	22.9
**Parents' level of education.**	**Paternal**		**Maternal**	
Scholarity				
No education	22	2.6%	23	2.7%
Elementary school (1rst to 4th class)	263	31%	316	37.2%
Junior high school (5th to 8th)	121	14.3%	128	15.1%
High school	155	18.3%	155	18.3%
Graduate	81	9.5%	100	11.8%
Missing data	207	24.4%	127	15%

^a^Classification of skin color taken from the interviewee's self-identification, according to the Brazilian Institute of Geography and Statistics – IBGE

## Discussion

ID is still one of the main health problems in the world with a prevalence of 1–3% depending on the study [3, 27] and a lifelong problem for the affected and their families. A previous survey, done in four schools for intellectually disabled maintained by the APAE in the mid-west of Santa Catarina, indicated that at least 15–20% of the students had other similar cases in the family (Maris, A.F., unpublished). Searching the literature, no epidemiological data to relate that finding was found, however, Fryns, J.P., that in earlier times published extensive diagnostic surveys in institutionalized populations with moderate to severe ID [28–32], estimated that up to 50% of those individuals had at least one additional case in the family (Fryns, J.P., personal communication in 2008). This data is virtually unknown, however urgently needed, because if familial cases have a high prevalence among the population of intellectually disabled individuals, the cause probably is genetic and the exact etiology essential to minimize recurrence. We reasoned it could be possible to identify most of the affected families of a region if we surveyed the special schools of that region, because of a high chance for at least one affected of the family attending.

To access the diagnostic status of the attendees and a possible familial recurrence, we included specific questions about family cases up to three generations in our questionnaire to gather not only the autosomal recessive or dominant cases, but also the X-linked and imprinted inheritance. The interview usually was a lengthy procedure that could take over 1 h, because the topic is emotional and many parents felt compelled to tell about their struggles, frustrations and hopes; and not a few had other cases in the family.

The receptivity of the special schools to the study varied. For a few it was a nuisance that they felt was disrupting their routine, because we needed them to establish contact with the parents and the interviews were made in school premises, but for most it was a welcome study and they were engaged to help. The teachers were always interested and when possible participated conducting or reviewing the questionnaires.

### Barriers encountered

Firstly, we considered that we could easily define three generations of the same family, trace a family tree and characterize all affected individuals, even if some of them were enrolled in different schools. The reality, though, was different, as we only had the identification (the complete names) of the participant and respective mother and father and, generally, the names of other affected family members who attended the same school. However, family relations were intricate; there were affected half-brothers of different fathers (and different surnames), or with the same father, but different mothers, often attending schools quite distant from one another, hindering the setting up of the family structure. As a result, the quality of the data was quite variable; family connections were often ignored, sometimes deliberately, due to family distancing. The complexity of trying to connect families of three generations from a large region, where one generation ago the average number of children was 5–12 (according to our survey), and including all affected cases of those three generations in one lineage, proved to be an impossible undertaking, because it connected too many families / individuals.

To follow a reproducible methodology, it was decided to research the general data by participant, regardless of kinship. Evidently this approach considers some families being represented more than once. For example, if two individuals from the same family (brothers or cousins) are participants, each was considered a family case. However, since three generations were considered (siblings, parents, uncles, cousins, grandparents and their siblings and their children – including deceased), most of the other affected members of the families were not participants.

### Lessons learnt

For 1.2% of the participants it was found that there were others (up to six) affected with ID in the family, with a similar presentation among them (raising the possibility a familial ID), however, not due to the same cause as the ID of the participant – thus classified as “Possibly familial but not linked to the participant” (Figs. 2 and 3d). This was unexpected and raises the question of how many unaffected people in the population have potential familial cases of ID in their extended (3 generation) family, nevertheless, a similar questionnaire applied to the general population probably would not retrieve data that is as rather informative as the one obtained here. Many of the participant parents told that they only became aware of the cases in the own family after having an affected child themselves. There is a stigma around the subject, even more pronounced when the disability is serious, syndromic, or more family members are affected. Apart from the sense of failure and often guilt, it can hurt the chances of family members to marry, so it is not something to talk about. That the “bad blood” came from one particular side of the family was an often-heard expression during the surveys.

### Male predominance

Among the 849 participants, over half were adults from 20 to 66 years of age (Fig. 1). The higher prevalence of males found is similar to other surveys of APAE schools in Brazil [33, 34]. The prevalence of approximately 30% more men diagnosed with ID is a known epidemiological fact [16, 34, 35], credited to X-linked conditions, which are believed to be responsible for more than half of hereditary familial ID [36]. In our study the sex ratio of the potential familial cases was 1.74 M:1F, compared to the 1.25 M:1F ratio found in sporadic cases (Fig. 3). However, only a fraction of the male excess of ID is attributable to mutations in genes located on the X chromosome and other factors, both genetic and non-genetic, are involved [37, 38]. For instance, Down syndrome, the most important cause of ID in the group of known etiologies in our study (13.7% - Table 1) had a sex ratio of 1.58 M:1F. Unlike the female predominance at birth in the general population, for Down syndrome more males are born [39], and there is a higher perinatal mortality in females with the syndrome [40]. Another important contributor for the overall male predominance in the present study were the 14.1% participants included in Autism Spectrum Disorders, where the sex ratio was 3.4 M:1F, similar to the 4 M:1F ratio reported for this diagnosis in a recent and extensive epidemiological survey from the Autism and Developmental Disabilities Monitoring Network in the United States [41]. Additionally, evidence indicates that females are far more resistant to mutational burden in neurodevelopmental genes than males, due to not well understood factors [42].

### Diagnostic situation

The diagnosis is the principle for all activities aimed at people with special needs, be it for early interventions, to improve motor, cognitive, emotional, social and language skills or for genetic counseling. In Brazil there are large differences in the diagnostic rates in special education schools [3, 33, 43]. The participants of this study and of other special need schools in Brazil are mainly individuals with moderate to severe and in some cases profound ID, since mild cases are integrated into regular schools with the aid of a special needs educator. The participants whose main clinical diagnostic is mild ID, Asperger syndrome, epilepsy, or CP, have wider difficulties than the main diagnostic reveals, related to cognition, communication, and/or adaptive skills. Of the 849 participants in our study, 521 (61.4%) did not have a defined etiological diagnosis and were classified into idiopathic cases (Table 1). CP was neither included as idiopathic nor as having a clear etiologic cause because, despite its high association with prematurity and hypoxic–ischaemic injury [44], CP is often related to congenital defects and possibly to a higher inherited genetic susceptibility to brain to injury after hypoxia [45, 46] or to underlying genetic diseases [47]. Indeed, several parents reported that their newborns had not suffered from hypoxia at birth (they cried as soon as they were born and were not cyanotic), we also found familial recurrence for CP allegedly caused by ischemia and, during the study, one of the students with the diagnosis of CP due to neonatal hypoxia was found to have Angelman syndrome (included in Table 1 as Angelman syndrome).

Solely 24.3% of the participants could be considered as having a well-defined etiologic diagnosis, 18.1% of genetic origin, most of them because of Down syndrome (13.7%), and 6.1% because of sequelae of infectious diseases (Table 1). The diagnostic status of special needs institutions in Brazil rarely is reported. When the diagnostic status of attendees of a special needs institution is only surveyed, defined etiological diagnoses are low, as shown in a survey done in the APAE of Itabira-MG, were of 289 students only 28 (9.6%) were found to have a defined etiological diagnosis (mainly Down syndrome) - for most the diagnosis was the degree of ID (mild, moderate and severe) [48]. On the other hand, at the APAE in Caxias do Sul, where researchers from the medical genetics service of the Hospital das Clínicas of Porto Alegre made a diagnostic effort, with chromosomal analysis, metabolic screening, imaging and other methods, the diagnostic rate was ~ 65, 47% of proved genetic etiology, (including 32% of the attendees of this school that had Down syndrome) [49]. However, no estimation of familial cases within this universe was made.

### Relevance of familial intellectual disability

Possibly the most relevant result of our survey was the collection of the family histories, which revealed that 34.5% of the participants represented potentially familial cases (Fig. 2b), a higher value than expected, with 18% mentioning four to 14 additional cases in the extended family (Fig. 2b, b1). Most familial cases are credited to X-linked conditions, in particular when affecting several generations and mainly affecting males, other causes include dominant conditions which often have incomplete penetrance and/or variable expressivity, and autosomal recessive conditions, which typically affect only siblings of the same brotherhood within a family, being more frequent in marriages among related parents. However, autosomal recessive conditions can recur in several generations when consanguineous marriages among a large family are frequent. Inbreeding families with several affected members have been an integral part of the determination of autosomal recessive genes. Studies carried out four to five decades ago, showed that in Brazil, consanguineous marriages presented very different distribution among states, ranging from 9% in the North and Northeast regions to 0.6% in the South [50].

The frequency of consanguineous marriages/unions among the parents of the participants of our study was 4% (Table 2), considerably higher than the 0.6% reported for the South region, suggesting autosomal recessive conditions. A recent study, that analyzed long contiguous stretches of homozygosity through chromosomal microarrays in patients with neurodevelopmental disorders in the State of Santa Catarina, suggested a descent from first to fifth grade for about 8.5% of them [51]. Even in a non-endogamous population, regional characteristics of immigration and marriages within the same ethnic group of immigrants can create endogamy pockets which favor reappearance of autosomal recessive disorders in several generations of a same family.

**The Brazilian population consists of four major groups:** local Indigenous; Portuguese, who colonized the country in the sixteenth century; Africans, brought from the 16th to the seventeenth century; and the various other European and Asian ethnic groups who immigrated to Brazil in the mid-nineteenth century [52, 53]. The state of Santa Catarina received mainly European immigrants. The first settlers were Portuguese, many of them originating from the Azores and, from the nineteenth century, immigrants started joining from other places, especially from Germany and Italy [54–56], rendering the state of Santa Catarina as the “whitest” of Brazil, with 84% of its citizens self-declaring as white, much above the national frequency of 47.7% [57]. This is reflected also in the about 80% of self-declared white and high proportion of European descendants found in our study (Table 2). Still, until recently there was a well-known resistance among Germans and Italians to marry outside their ethnic groups and religious beliefs, especially in the rural region of Santa Catarina, favoring endogamy. Families with low levels of education and living in rural communities, more isolated from large centers, usually have large family groups, with extensive brotherhoods [8, 58]. This, and the low educational level of the parents, where about half have not attended high school (Table 2), can explain the high number of affected in some families.

**External validation:** Previous research by A. Maris at special schools from another region of Santa Catarina had revealed that familial intellectual disability could play an important role within the universe of the intellectually disabled, which was confirmed here. The questionnaire (available as Supplementary material) was designed to minimize the bias of having different interviewers; The survey contained directed questions, about phenotypic and developmental characteristics and repeated questions about the presence of other similar cases for each generation accessed, to collect data with greater reliability; Interviewers were previously trained to understand the relevance of dysmorphology and potential inheritance; Families were approached in different ways, some spontaneously attended the interview, most, however, were recruited when they came to school for different reasons. With a few exceptions, all those directly approached agreed to sign the consent form and to participate.

The family history approach of our survey might not lead to similar results in a typical population of intellectually disabled of industrialized countries, where the families are small and number of children per couple is only about one or two. However, the observations made by J.P. Fryns, although outdated, reinforce the relevance of familial ID within the universe of individuals with moderate to severe ID, also in Europe. Our sample gives a unique view into the diagnostic situation and the relevance of familial cases in intellectually disabled in Brazil, and might well represent the situation of most developing countries where often the families are large and the means to conduct molecular testing is not widely available, as could be seen by the diagnostic status of the students of our study.

## Conclusion

This study highlights the low frequency (24 to 35% when including CP as caused by oxygen deprivation at birth) of students whose ID has a known etiology, showing that there is a large margin for improvement for causal diagnoses. Most reported diagnoses are limited to the description of the main symptom of the affected individual, e.g., mild, moderate or severe intellectual disability. We also uncover the high proportion of possibly familial cases (34.5%) in a population with relatively few consanguineous marriages. The high frequency of parents with low level of education, and therefore low socioeconomic means, may explain the low percentage of etiologic diagnoses and the high number of affected within one extended family. Considering the social and economic cost of ID, and the fact that ID is not curable, our results call for public health actions directed to this population for adequate diagnoses, giving access to molecular testing and genetic counseling to enable prevention, because recurrence of ID in a family is not a rare event.

## Supplementary information

**Additional file 1:** Questionnaire.

## Data Availability

The data set of this study will not be publicly available, in accordance with the ethical guidelines of the human research ethics committee of the Hospital Joana de Gusmão. The data in this study will be used exclusively for research purposes approved by the committee.

## Abbreviations

APAE: Associação de Pais e Amigos dos Excepcionais (Association of parents and friends of the exceptional)
CP: Cerebral Palsy
FCEE: Fundação Catarinense de Educação Especial (Catarinense Foundation of Special Education)
ID: Intellectual Disability
IQ: Intelligence Quotient
UFSC: Santa Catarina Federal University
