# Supplementary material for: Etiology of intellectual disability in individuals from special education schools in the south of Brazil
Source: BMC Pediatr. 2020 Nov 4;20:506. doi: 10.1186/s12887-020-02382-5 (PMC7640392; doi:10.1186/s12887-020-02382-5)
Supplement: Supplementary file 1 — Additional file 1: Questionnaire. [file 12887_2020_2382_MOESM1_ESM.pdf]

Federal University of Santa Catarina
Center of Biological Sciences
Department of Cell Biology, Embryology and Genetics - BEG
Development Neurogenetics Laboratory

LABORATÓRIO DE NEUROGENÉTICA DO DESENVOLVIMENTO

QUESTIONNAIRE - SPECIAL SCHOOLS

TRANSLATED VERSION

DATE: ___/___/____

SCHOOL____________________________ INTERVIEWER____

NAME OF THE RESPONDENT____/ __MOTHER __FATHER__OTHER

COMMENTS:____

____

PERSONAL DATA:

STUDENT'S NAME:____

AGE: _____________ GENDER:___ MALE __ FEMALE DATE OF BIRTH:______________________________

SKIN COLOR: __BLACK __YELLOW __ BROWN __WHITE / ADOPTIVE: __NO __YES __DOES NOT WANT TO RESPOND

CITY / STATE OF BIRTH:____

ADDRESS:____

CITY: ____ CONTACT PHONES: ___________________________

RESPONSIBLE: __MOTHER __FATHER __OTHER:____

DIFFICULTY / DIAGNOSIS: ____

____ PERCEIVED AT WHAT AGE: ______________

WALKS: __NO __YES. SINCE WHAT AGE? ___________ HAS A GOOD BALANCE: __YES __NO

SPEAKS: ___VERY WEL / ___ WELL / __ IS NOT VERY COMPREHENSIVE __DOES NOT SPEAK

FROM WHAT AGE ON? _________

CONVULSION: __NO / __ONE/FEW / __VARIOUS/MANY / __CONTROLLED WITH MEDICATION

AGE AT THE FIRST: ______

VISION: __GOOD __WEAK __BLIND HEARING: __GOOD __WEAK __DEAFNESS

COMMENTS:____

____

PHYSICAL CHARACTERISTICS THAT ARE DIFFERENT: ___ NONE / __HEAD / __ EYES / __MOUTH / __NOSE / __EAR

__HAND / __FOOT / __GENITAL / __OTHER/ EXPLAIN: ____

____---------------_______________________

NEEDS HELP TO EAT: __YES __NO NEEDS HELP TO DRESS: __YES __NO TO BATHI: __YES __NO

READS: __WELL __LITTLE __NO / WRITES: __WELL __LITTLE __NO CAN COUNT: __WELL __LITTLE __NO

COMMENTS:____

____

FAMILY DATA:

NAME OF THE FATHER:____

CITY / STATE OF BIRTH: ____

ORIGIN: __GERMAN __ITALIAN __PORTUGUESE __AFRICAN __INDIGENOUS __BRAZILIAN __OTHER

PROFESSION: ___________________________________ SCHOOLING: ______________________________

NAME OF THE MOTHER____

CITY / STATE OF BIRTH: ____

ORIGIN: __GERMAN __ITALIAN __PORTUGUESE ___AFRICAN __INDIGENOUS __BRAZILIAN __OTHER

PROFESSION: _________________________________ SCHOOLING: ________________________________

HAS THE STUDENT SIBLINGS? __NO __YES

HOW MANY HAVE THE SAME MOTHER **AND** THE SAME FATHER?: BROTHERS ____; SISTERS _____

HALF BROTHERS BY FATHER: BOYS ___; GIRLS ___.

HALF-BROTHERS BY THE MOTHER: BOYS ___; GIRLS ____.

FAMILY HISTORY - *ONLY RELEVANT FOR THE STUDENT'S BIOLOGICAL (BLOOD) RELATIVES*

__ DOES NOT KNOW THE STUDENT'S BIOLOGICAL FAMILY

__ DOES NOT KNOW THE FAMILY OF THE FATHER __ DOES NOT KNOW THE FAMILY OF THE MOTHER

1- DO ANY OF THE **STUDENT'S PARENTS** OR **BROTHERS** HAVE ANY DIFFICULTY SIMILAR TO THE STUDENT?

__NO __YES

FATHER __ MOTHER ___ BROTHER(S)* ___ SISTER(S)* ____ * Number of brothers or sisters with the difficulty

TYPE OF DIFFICULTIES:

____

____

HALF-BROTHERS BY FATHER: BOYS ___; GIRLS ___.

HALF-BROTHERS BY THE MOTHER: BOYS ___; GIRLS ___.

TYPE OF DIFFICULTIES:

____

____

2 - DO SOME OF THE STUDENT'S **GRANDPARENTS** HAVE ANY DIFFICULTY SIMILAR TO THE STUDENT? __NO

__YES, FROM THE MOTHER'S SIDE:

__GRANDMOTHER __GRANDFATHER

__SISTER(S) OF GRANDMA __BROTHER(S) OF GRANDMA

__SISTER(S) OF GRANDFATHER __BROTHER(S) OF GRANDFATHER

TYPE OF DIFFICULTIES:

____

____

__YES - BY THE FATHER'S SIDE:

__GRANDMOTHER __GRANDFATHER

__SISTER(S) OF GRANDMA __ BROTHER(S) OF GRANDMA

__SISTER(S) OF GRANDFATHER __BROTHER(S) OF GRANDFATHER

TYPE OF DIFFICULTIES:

____

____

3 - DO SOME OF THE STUDENTS UNCLES/AUNTS HAVE INTELLECTUAL DISABILITY OR DIFFICULTY SIMILAR TO THE STUDENT? ___NO

___YES - MOTHER'S BROTHERS*: UNCLE(S) ___ AUNT(S) ___

YES, FATHER'S BROTHERS*: UNCLE(S) ___ AUNT(S) ___

* number of uncles or aunts with disability/difficulty

TYPE OF DIFFICULTIES:

____

____

TRANSLATED VERSION

4 - DO ANY OF THE STUDENTS COUSINS HAVE MENTAL DISABILITY OR DIFFICULTY LIKE THE STUDENT? ___NO

___YES, MOTHER'S SISTER CHILDREN: COUSINS (MALES)* ___ and COUSINS (FEMALES)* ___

___YES, MOTHER'S BROTHERS CHILDREN: COUSINS (MALES)* ___ and COUSINS (FEMALES)* ___

___YES, CHILDREN OF SISTERS OF THE FATHER: COUSINS (MALES) ___ and COUSINS (FEMALES)* ___

___ YES, CHILDREN OF BROTHERS OF THE FATHER: COUSINS (MALES)* ___ and COUSINS (FEMALES)* ___

* Put the number of cousins or cousins with problem in the lines above

TYPE OF DIFFICULTIES:

6 - SOME OF THE STUDENT'S PARENTS OR BROTHERS HAVE OR HAD SERIOUS LEARNING DIFFICULTIES AT SCHOOL OR TO UNDERSTAND MORE DIFFICULT THINGS? ___NO

___FATHER ___MOTHER ___BROTHER(S) ___SISTER(S)

HALF-BROTHERS BY THE FATHER: BOYS* ___; GIRLS* ___.

HALF-BROTHERS BY MOTHER: BOYS* ___; GIRLS*___.

* Put the number of brothers or sisters with a problem on the line

COMMENTS:

7 - STUDENT PARENTS ARE RELATED? : ___NO ___YES

IF THEY ARE, WHAT ARE THEIR RELATIVES:

8 - WHE THE STUDENT WAS BORN, WAS HE TESTED IN THE NEWBORN SCREENING?

___NO ___YES

____YES, AND A PROBLEM WAS DETECTED. DO YOU KNOW WHAT IS WAS?

9 - WAS THERE ANY GENETICS CHROMOSOME TESTING (KARYOTYPE)? ___NO ___YES, NORMAL

YES, WITH A ABNORMALITY - WHICH? ____

10 - WERE THERE PROBLEMS DURING PREGNANCY?

___NO ___HIGH FEVER ___RUBELLA ___TOXOSPLASMOSIS ___DIABETES ___A TUMBLE ___CYTOMEGALOVIRUS

___CONVULSION ___ALCOHOL ___MEDICATIONS ___ILLICIT DRUGS ___MISSTREATMENT (PHYSICAL ABUSE)

___OTHERS/ EXPLAIN:

11 – THE BIRTH OF THE CHILD WAS: :

___PRE-MATURE ___ AT TERM ___PASSED THE TIME

___VERY COMPLICATED ___NORMAL ___FORCEPS ___CESAREAN

EXPLAIN:

TRANSLATED VERSION

12 - THE BABY? :

___CRYED SOON ___HAD OXYGEN DEPRIVATION AT BIRTH ___HAD SOME PROBLEM AS SOON AS IT WAS BORN

EXPLAIN:

13 - WHAT DO YOU BELIEVE TO BE THE CAUSE FOR THE DIFFICULTIES OF YOUR SON?

14 - NOTE BELOW IF YOU HAVE ANY OTHER INFORMATION YOU CONSIDER IMPORTANT:

TRANSLATED VERSION
